# Supplementary material for: Heparin-based hydrogel scaffolding alters the transcriptomic profile and increases the chemoresistance of MDA-MB-231 triple-negative breast cancer cells
Source: Biomater Sci. 2020 Feb 13;8(10):2786–96. doi: 10.1039/c9bm01481k (PMC7497406; doi:10.1039/c9bm01481k)
Supplement: Supplementary file 2 [file BM-008-C9BM01481K-s002.zip › Supplementary File 4/HP-B hydrogel + EGF (s)vControl/Pathways/my_analysis.Gsea.1545201170987/HALLMARK_PI3K_AKT_MTOR_SIGNALING.html]

Details for gene set HALLMARK\_PI3K\_AKT\_MTOR\_SIGNALING[GSEA]

|  || Dataset | expr.class.cls#HYDEGF\_versus\_CONTROL.class.cls#HYDEGF\_versus\_CONTROL\_repos |
| Phenotype | class.cls#HYDEGF\_versus\_CONTROL\_repos |
| Upregulated in class | CONTROL |
| GeneSet | HALLMARK\_PI3K\_AKT\_MTOR\_SIGNALING |
| Enrichment Score (ES) | -0.32163563 |
| Normalized Enrichment Score (NES) | -1.3774464 |
| Nominal p-value | 0.019900497 |
| FDR q-value | 0.046469133 |
| FWER p-Value | 0.49 |
Table: GSEA Results Summary

  

Fig 1: Enrichment plot: HALLMARK\_PI3K\_AKT\_MTOR\_SIGNALING      
 Profile of the Running ES Score & Positions of GeneSet Members on the Rank Ordered List

  

| PROBE | DESCRIPTION (from dataset) | GENE SYMBOL | GENE\_TITLE | RANK IN GENE LIST | RANK METRIC SCORE | RUNNING ES | CORE ENRICHMENT || 1 | E2F1 | na |  |  | 358 | 2.297 | 0.0128 | No |
| 2 | TRAF2 | na |  |  | 932 | 1.730 | 0.0066 | No |
| 3 | EGFR | na |  |  | 1395 | 1.470 | 0.0026 | No |
| 4 | RAF1 | na |  |  | 1584 | 1.399 | 0.0120 | No |
| 5 | NOD1 | na |  |  | 1782 | 1.335 | 0.0200 | No |
| 6 | MKNK1 | na |  |  | 1901 | 1.293 | 0.0316 | No |
| 7 | HRAS | na |  |  | 2033 | 1.257 | 0.0420 | No |
| 8 | RPS6KA1 | na |  |  | 2077 | 1.244 | 0.0569 | No |
| 9 | RPTOR | na |  |  | 2141 | 1.227 | 0.0704 | No |
| 10 | MAP2K3 | na |  |  | 2199 | 1.211 | 0.0841 | No |
| 11 | AKT1 | na |  |  | 2221 | 1.205 | 0.0995 | No |
| 12 | PLCG1 | na |  |  | 2414 | 1.148 | 0.1052 | No |
| 13 | DDIT3 | na |  |  | 2748 | 1.066 | 0.1025 | No |
| 14 | PAK4 | na |  |  | 2949 | 1.017 | 0.1060 | No |
| 15 | ITPR2 | na |  |  | 3448 | 0.914 | 0.0924 | No |
| 16 | CDK2 | na |  |  | 3627 | 0.880 | 0.0952 | No |
| 17 | TSC2 | na |  |  | 4032 | 0.807 | 0.0852 | No |
| 18 | GSK3B | na |  |  | 5099 | 0.638 | 0.0381 | No |
| 19 | PIK3R3 | na |  |  | 5161 | 0.627 | 0.0436 | No |
| 20 | TRIB3 | na |  |  | 5408 | 0.590 | 0.0388 | No |
| 21 | STAT2 | na |  |  | 6213 | 0.474 | 0.0032 | No |
| 22 | ARHGDIA | na |  |  | 6680 | 0.413 | -0.0155 | No |
| 23 | MAPK8 | na |  |  | 6958 | 0.378 | -0.0248 | No |
| 24 | AP2M1 | na |  |  | 7100 | 0.358 | -0.0272 | No |
| 25 | MAPKAP1 | na |  |  | 8358 | 0.201 | -0.0903 | No |
| 26 | FGF17 | na |  |  | 8562 | 0.179 | -0.0984 | No |
| 27 | PPP2R1B | na |  |  | 8888 | 0.137 | -0.1136 | No |
| 28 | IRAK4 | na |  |  | 8918 | 0.132 | -0.1133 | No |
| 29 | SFN | na |  |  | 9071 | 0.119 | -0.1196 | No |
| 30 | CAMK4 | na |  |  | 9137 | 0.111 | -0.1215 | No |
| 31 | MAPK9 | na |  |  | 9176 | 0.105 | -0.1220 | No |
| 32 | NFKBIB | na |  |  | 9368 | 0.079 | -0.1309 | No |
| 33 | MAPK10 | na |  |  | 9680 | 0.052 | -0.1465 | No |
| 34 | ACACA | na |  |  | 9794 | 0.037 | -0.1519 | No |
| 35 | PRKAR2A | na |  |  | 9800 | 0.035 | -0.1517 | No |
| 36 | CAB39 | na |  |  | 11189 | -0.121 | -0.2226 | No |
| 37 | PIN1 | na |  |  | 11988 | -0.215 | -0.2614 | No |
| 38 | MKNK2 | na |  |  | 12029 | -0.222 | -0.2605 | No |
| 39 | CDK4 | na |  |  | 12236 | -0.251 | -0.2678 | No |
| 40 | GRB2 | na |  |  | 12300 | -0.261 | -0.2675 | No |
| 41 | AKT1S1 | na |  |  | 12408 | -0.273 | -0.2694 | No |
| 42 | RALB | na |  |  | 12591 | -0.296 | -0.2748 | No |
| 43 | MYD88 | na |  |  | 12655 | -0.306 | -0.2739 | No |
| 44 | PPP1CA | na |  |  | 12980 | -0.337 | -0.2863 | No |
| 45 | SLC2A1 | na |  |  | 13117 | -0.356 | -0.2885 | No |
| 46 | ECSIT | na |  |  | 13198 | -0.371 | -0.2876 | No |
| 47 | PIKFYVE | na |  |  | 13266 | -0.381 | -0.2859 | No |
| 48 | PTPN11 | na |  |  | 13310 | -0.388 | -0.2828 | No |
| 49 | PFN1 | na |  |  | 13723 | -0.445 | -0.2982 | No |
| 50 | PRKAA2 | na |  |  | 13965 | -0.483 | -0.3042 | No |
| 51 | SQSTM1 | na |  |  | 13980 | -0.486 | -0.2983 | No |
| 52 | SMAD2 | na |  |  | 14019 | -0.491 | -0.2935 | No |
| 53 | DUSP3 | na |  |  | 14447 | -0.548 | -0.3083 | No |
| 54 | THEM4 | na |  |  | 14536 | -0.561 | -0.3052 | No |
| 55 | PDK1 | na |  |  | 14692 | -0.588 | -0.3053 | No |
| 56 | TBK1 | na |  |  | 14771 | -0.599 | -0.3011 | No |
| 57 | RIPK1 | na |  |  | 14875 | -0.615 | -0.2981 | No |
| 58 | CDKN1A | na |  |  | 14882 | -0.616 | -0.2899 | No |
| 59 | YWHAB | na |  |  | 15452 | -0.711 | -0.3099 | No |
| 60 | CDKN1B | na |  |  | 15677 | -0.758 | -0.3112 | Yes |
| 61 | CFL1 | na |  |  | 15790 | -0.783 | -0.3063 | Yes |
| 62 | CDK1 | na |  |  | 15956 | -0.811 | -0.3038 | Yes |
| 63 | TNFRSF1A | na |  |  | 16016 | -0.823 | -0.2956 | Yes |
| 64 | CLTC | na |  |  | 16121 | -0.850 | -0.2894 | Yes |
| 65 | ATF1 | na |  |  | 16159 | -0.860 | -0.2795 | Yes |
| 66 | MAP3K7 | na |  |  | 16187 | -0.867 | -0.2690 | Yes |
| 67 | TIAM1 | na |  |  | 16222 | -0.879 | -0.2587 | Yes |
| 68 | MAPK1 | na |  |  | 16445 | -0.935 | -0.2575 | Yes |
| 69 | PTEN | na |  |  | 16477 | -0.941 | -0.2462 | Yes |
| 70 | UBE2D3 | na |  |  | 16494 | -0.946 | -0.2340 | Yes |
| 71 | PLA2G12A | na |  |  | 16733 | -1.006 | -0.2327 | Yes |
| 72 | UBE2N | na |  |  | 16783 | -1.022 | -0.2212 | Yes |
| 73 | RIT1 | na |  |  | 16839 | -1.039 | -0.2098 | Yes |
| 74 | ARF1 | na |  |  | 16848 | -1.042 | -0.1959 | Yes |
| 75 | NGF | na |  |  | 16886 | -1.050 | -0.1834 | Yes |
| 76 | CXCR4 | na |  |  | 17068 | -1.103 | -0.1777 | Yes |
| 77 | EIF4E | na |  |  | 17315 | -1.190 | -0.1743 | Yes |
| 78 | NCK1 | na |  |  | 17360 | -1.202 | -0.1600 | Yes |
| 79 | ACTR2 | na |  |  | 17574 | -1.284 | -0.1536 | Yes |
| 80 | PRKAG1 | na |  |  | 17624 | -1.307 | -0.1382 | Yes |
| 81 | CALR | na |  |  | 17688 | -1.326 | -0.1232 | Yes |
| 82 | CSNK2B | na |  |  | 17721 | -1.343 | -0.1065 | Yes |
| 83 | RPS6KA3 | na |  |  | 17760 | -1.363 | -0.0897 | Yes |
| 84 | RAC1 | na |  |  | 17921 | -1.452 | -0.0782 | Yes |
| 85 | CAB39L | na |  |  | 18065 | -1.523 | -0.0647 | Yes |
| 86 | ACTR3 | na |  |  | 18277 | -1.659 | -0.0530 | Yes |
| 87 | PITX2 | na |  |  | 18546 | -1.861 | -0.0414 | Yes |
| 88 | HSP90B1 | na |  |  | 19003 | -2.588 | -0.0298 | Yes |
| 89 | ARPC3 | na |  |  | 19099 | -2.905 | 0.0052 | Yes |
Table: GSEA details [plain text format]

  

Fig 2: HALLMARK\_PI3K\_AKT\_MTOR\_SIGNALING      
 Blue-Pink O' Gram in the Space of the Analyzed GeneSet

  

Fig 3: HALLMARK\_PI3K\_AKT\_MTOR\_SIGNALING: Random ES distribution      
 Gene set null distribution of ES for **HALLMARK\_PI3K\_AKT\_MTOR\_SIGNALING**
